# Anti-GD2-ch14.18/CHO coated nanoparticles mediate glioblastoma (GBM)-specific delivery of the aromatase inhibitor, Letrozole, reducing proliferation, migration and chemoresistance in patient-derived GBM tumor cells

**DOI:** 10.18632/oncotarget.15073

**Published:** 2017-02-03

**Authors:** Amanda Tivnan, Tatjana Heilinger, Joanne M Ramsey, Gemma O’Connor, Jenny L Pokorny, Jann N Sarkaria, Brett W Stringer, Bryan W Day, Andrew W Boyd, Ella L Kim, Holger N Lode, Sally-Ann Cryan, Jochen H.M Prehn

**Affiliations:** ^1^ Centre for Systems Medicine, Department of Physiology and Medical Physics, Royal College of Surgeons in Ireland, York House, Dublin 2, Ireland; ^2^ IMC Fachhochschule Krems, University of Applied Sciences, Krems, Austria; ^3^ School of Pharmacy, Royal College of Surgeons in Ireland, York House, Dublin 2, Ireland & Tissue Engineering Research Group, Department of Anatomy, RCSI and Centre for Research in Medical Devices (CURAM), NUIG, Ireland; ^4^ Department of Radiation Oncology, Mayo Clinic, Rochester, MN, United States of America; ^5^ Department of Neurosurgery, Stanford University, Stanford, CA 94305, USA; ^6^ Brain Cancer Research Unit, QIMR Berghofer Medical Research Institute, Brisbane, Australia; ^7^ Laboratory of Neurooncology, Department of Neurosurgery, Johannes Gutenberg University Medical Center, Mainz, Germany; ^8^ Department of Paediatrics and Paediatric Haematology/Oncology, University of Greifswald, Greifswald, Germany

**Keywords:** glioblastoma, nanoparticles, aromatase inhibitor, miRNA-191, brain

## Abstract

Aromatase is a critical enzyme in the irreversible conversion of androgens to oestrogens, with inhibition used clinically in hormone-dependent malignancies. We tested the hypothesis that targeted aromatase inhibition in an aggressive brain cancer called glioblastoma (GBM) may represent a new treatment strategy. In this study, aromatase inhibition was achieved using third generation inhibitor, Letrozole, encapsulated within the core of biodegradable poly lactic-co-glycolic acid (PLGA) nanoparticles (NPs). PLGA-NPs were conjugated to human/mouse chimeric anti-GD2 antibody ch14.18/CHO, enabling specific targeting of GD2-positive GBM cells. Treatment of primary and recurrent patient-derived GBM cells with free-Letrozole (0.1 μM) led to significant decrease in cell proliferation and migration; in addition to reduced spheroid formation. Anti-GD2-ch14.18/CHO-NPs displayed specific targeting of GBM cells in colorectal-glioblastoma co-culture, with subsequent reduction in GBM cell numbers when treated with anti-GD2-ch14.18-PLGA-Let-NPs in combination with temozolomide. As miR-191 is an estrogen responsive microRNA, its expression, fluctuation and role in Letrozole treated GBM cells was evaluated, where treatment with premiR-191 was capable of rescuing the reduced proliferative phenotype induced by aromatase inhibitor. The repurposing and targeted delivery of Letrozole for the treatment of GBM, with the potential role of miR-191 identified, provides novel avenues for target assessment in this aggressive brain cancer.

## INTRODUCTION

Glioblastoma (GBM) is the most lethal form of brain tumor and the most prevalent malignant primary brain tumor in adults. This highly aggressive tumor has not only a poor prognosis and treatment limitations, but also direct repercussions on quality of life and cognitive functions of the patient. With 3.19 in 100,000 people newly diagnosed each year GBM has the highest number of cases of all malignant brain tumors [1]. Until recently, the World Health Organization (WHO) classified gliomas into grades I to IV defined by increasing degrees of undifferentiated phenotype, grade of malignancy, proliferative index, response to treatment and survival time. While Grade I and II described benign and relatively non-malignant tumors, Grade III were tumors of low-grade malignancy, and Grade IV astrocytoma were designated the term glioblastoma (GBM) [2]. In 2016, CNS tumors were redefined by the WHO to incorporate both histological and molecular features including isocitrate dehydrogenase 1 (IDH)-wildtype, IDH-mutant, giant cell glioblastoma, gliosarcoma, epithelioid GBM and diffuse midline glioma, H3 K27 mutant [3]. Unfortunately however the median survival rate for GBM remains at 14.6 months [4], with a five year survival rate of only 4–5%. This dismal outcome motivates a search for novel and effective therapies to treat this incurable disease.

A combination of resection, radiotherapy (RT) and chemotherapy is the current standard of care for treating GBM. The degree of resection is of greatest importance as it can prolong survival significantly [5–7]; however, it is difficult to achieve complete resection without causing neurological deficits due to the highly invasive capacity of GBM cells in infiltrating adjacent brain tissue [8]. Ionizing radiation therapy remains the most clinically effective treatment strategy for most CNS tumors. First-line chemotherapy for GBM is the DNA alkylating agent temozolomide (TMZ). Despite the fact that several chemotherapies are available, patients treated with TMZ have the highest median survival rate compared to other chemotherapeutic agents [4, 9]. TMZ has much less toxicity than other chemotherapeutics and is administered orally.

Although the number of studies of GBM has grown and current therapy regimes have improved, only little improvement has been achieved in the last decade, with patients still waiting for a breakthrough which extends life beyond the median 14 months of survival post initial diagnosis. Unfortunately the majority of preclinical therapies which were shown to be effective have subsequently failed in the clinic. A promising approach, which holds real translation potential for the treatment of GBM, is drug repurposing [10–12].

Aromatase, or estrogen synthase, is a critical enzyme in the irreversible conversion of androgens to estrogen [13]. The aromatase enzyme is comprised of a microsomal cytochrome P450 complex which is a product of the *CYP19A1* gene. Innate aromatase activity is found to be present in gonadal tissues, uterus, breast, prostate, epididymis, placenta, adrenal glands, liver, skin, muscle, vascular endothelium, bone and brain [14]. Moreover estrogen is associated with several cancers and protects against adverse symptoms during the peri- and postmenopausal intervals, stimulating cellular proliferation, migration and growth of reproductive tissues [15]. The inhibition of aromatase enzyme is currently used in the treatment of hormone-dependent breast cancer, alterations of ovarian and endometrial function and treatment of benign disorders like gynecomastia as uncontrolled proliferation is targeted. In the present study, we therefore investigated the effects of aromatase inhibition on GBM cells proliferation, migration and, ultimately when used as an adjunct therapy, chemoresistance.

Specifically targeting drugs to disease sites within targeted polymeric carriers offers great potential to eliminate adverse side effects. Poly lactic-*co*-glycolic acid (PLGA) is a well-established polymeric excipient (FDA approved) consisting of poly lactic acid (PLA) and poly glycolic acid (PGA). Disialogangliosidase2 (GD2) is a highly glycosylated sphingolipid which has been shown to be highly expressed in high-grade gliomas [16] in addition to several other cancer types [17]. It was also reported as a target for glioblastoma [18] but, to date, there has been no clinical development related to GBM. Clinical experience with anti-GD2 antibodies was generated in neuroblastoma, a paediatric malignancy characterized by high GD2 expression. Several anti-GD2 antibodies are under clinical development and dinutuximab (ch14.18 produced in SP2/0 cells) was approved for the treatment of neuroblastoma in combination with IL2 and GM-CSF [19]. In Europe, ch14.18 was remanufactured in CHO cells (anti-GD2-ch14.18/CHO) [20, 21], which is currently used in clinical trials [22, 23]. Based on these considerations, we investigated whether ch14.18/CHO may provide a novel approach to target nanoparticles to glioblastoma and thereby minimise off-target effects.

In this study, biodegradable PLGA nanoparticles containing the aromatase inhibitor, Letrozole, were synthesized and capable of selective target recognition in co-culture experiments. Furthermore it was determined that aromatase inhibition lead to improved drug response to clinically relevant chemotherapeutic treatment.

## RESULTS

### Assessment of aromatase expression

**Figure 1:**
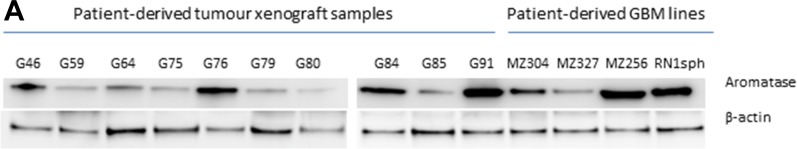
Evaluation of aromatase expression Aromatase (Cytochrome P450) protein expression was assessed in a panel of patient-derived GBM tumor xenograft samples and cell lines. Isolated protein lysates were provided from patient-derived glioblastoma xenografts (G46, G59, G64, G76, G79, G80, G84, G85 and G91) by the Mayo Clinic Brain Tumor SPORE [24–27]. These lysates, in addition to primary (MZ-327 and RN1) and recurrent (MZ-256 and MZ-304) GBM patient-derived cell lines were assessed by Western blotting for aromatase expression (50–55 kDa, Abcam, 1:250) with β-actin (1:5000) as a loading control. All clinical data pertaining to patient-derived glioblastoma xenograft tumor samples is provided in Supplementary Table 1.

To evaluate the expression of aromatase as a target in patient-derived glioblastoma cells we investigated the protein levels of P450 (aromatase) in a panel of both patient-derived cell lines and xenograft tumor lysates. Glioblastoma cell lines derived from primary tumors, MZ-327 and RN1 and recurrent derived GBM tumors, MZ-256 and MZ-304, in addition to several patient derived xenograft tumour lysates provided by the Mayo Clinic Brain Tumor SPORE (G46, G59, G64, G75, G76, G79, G80, G84, G85 and G91, Supplementary Table 1) [24–27] were assessed by Western blot (Figure 1). Notably, all GBM samples assessed express P450 at the molecular weight of 50–55 kDa, corresponding to the apparent molecular weight of aromatase.

### The effects of Letrozole treatment on GBM migration, proliferation and spheroid formation

**Figure 2:**
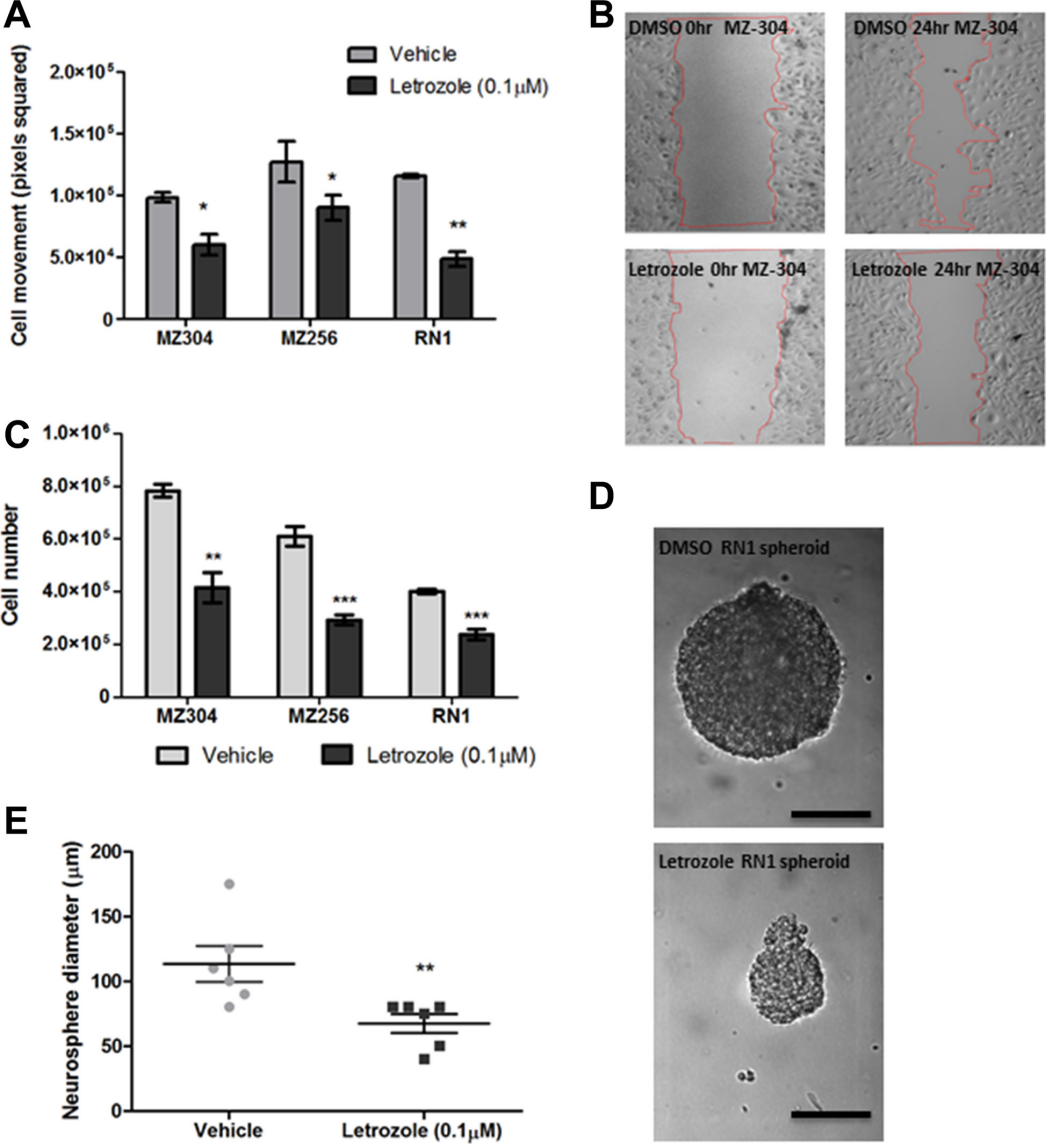
Assessment of the effects of Letrozole treatment on GBM migration, proliferation and spheroid formation Primary (RN1) and recurrent (MZ-256 and MZ-304) GBM cell lines were incubated with Letrozole (0.1 μM) for 24–72 hours, to determine if the cellular function of GBM cell lines changes upon treatment with the aromatase inhibitor, Letrozole. Notably all cells treated with Letrozole showed a significant decrease of cell movement compared to vehicle-treated (DMSO) controls (**A** and **B**). As shown (**C**), cell number was significantly reduced in all three cell lines when treated with Letrozole (0.1 μM) for 72 hours and RN1luc cells displayed reduced spheroid formation capabilities when exposed to Letrozole (0.1 μM) over a 72 hour period (**D** and **E)**, *n* = 3 mean ± SEM, *p < 0.05, **p < 0.01, ***p < 0.001).

To address the role of aromatase in GBM cells, the effects of P450-specific inhibitor, Letrozole, was further assessed. Primary (RN1) and recurrent (MZ-256 and MZ-304) GBM cell lines were incubated with Letrozole (0.1 μM) for 24–72 hours, to determine if the Letrozole has an impact on biological characteristics of GBM cell lines such as motility, proliferation and sphere-forming potential. Notably all cells treated with Letrozole showed a significant decrease in cell motility compared to vehicle-treated (DMSO) controls (Figure 2A and 2B). As shown in Figure 2C, cell number was significantly reduced in all three cell lines when treated with Letrozole (0.1 μM) for 72 hours and RN1 cells displayed reduced spheroid formation capabilities when exposed to Letrozole (0.1 μM) over a 72 hour period (Figure 2D and 2E *p < 0.05, **p < 0.01, ***p < 0.001).

### Assessment of GD2 expression in GBM cells

**Figure 3:**
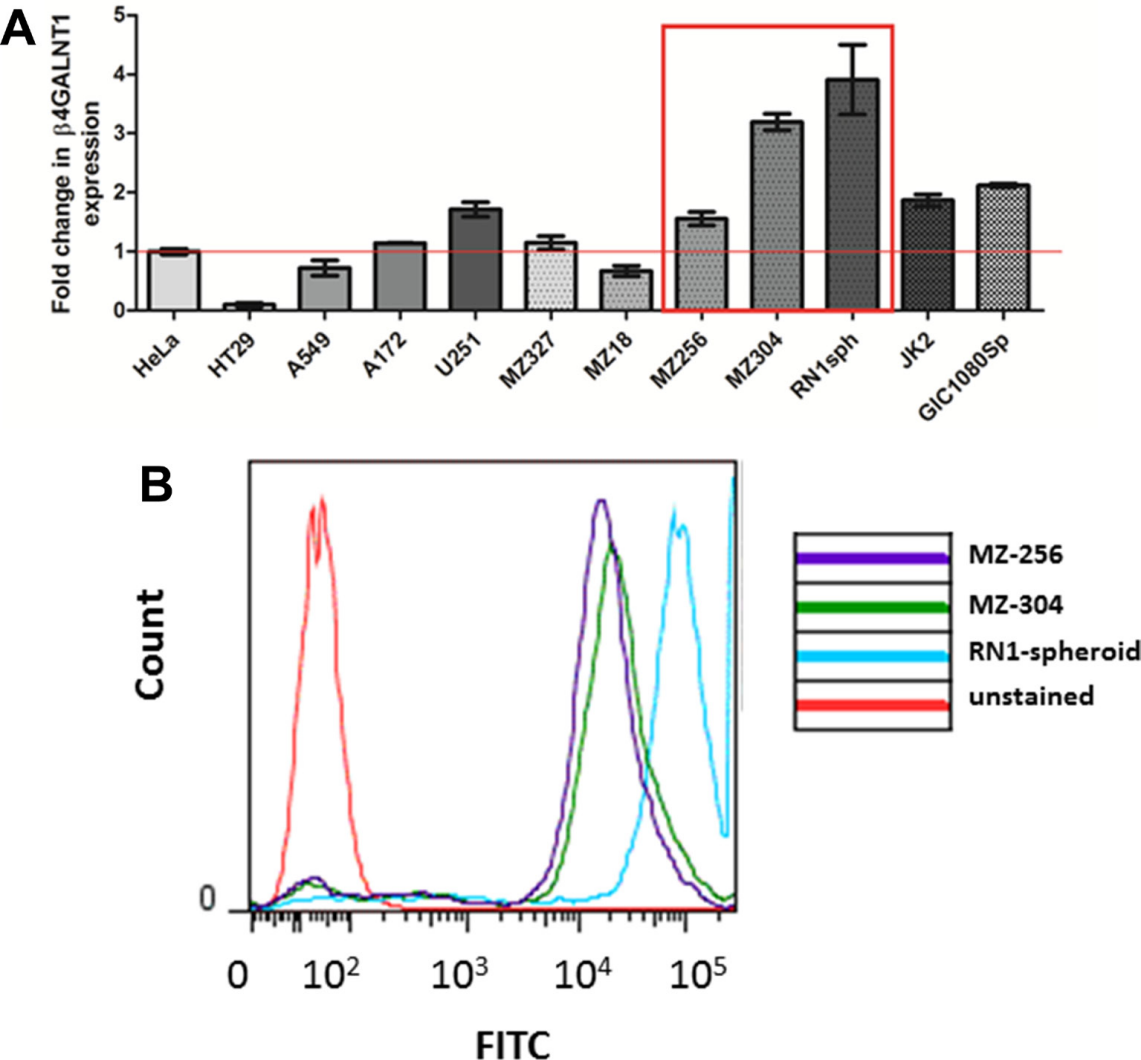
GD2 expression in GBM and non-GBM cell lines In order to carry out a preliminary screen of a panel of both GBM and non-GBM cell lines, qualitative PCR (qPCR) was carried out to asses mRNA expression of the key enzyme responsible for the synthesis of disialogangliosidase 2 (GD2). The expression levels of β1,4-N-acetylgalactosaminyltransferase (*β4GANT1)* mRNA transcript was assessed in *n* = 2 non-GBM lines (HT29 and A549), *n* = 2 commercially available GBM lines (A152 and U251), *n* = 4 primary GBM-patient derived lines (MZ-327, MZ-18, RN1spheroid and JK2), *n* = 2 recurrent GBM lines (MZ-256 and MZ-304) and a glioma initiating cell line (GIC1080Sp) relative to the cervical cancer cell line, HeLa. As shown (**A**) RN1spheroid, MZ-256 and MZ-304 have greatest *β4GANT1* expression. These highlighted lines were assessed by flow cytometry using a GD2-FITC tagged primary antibody (**B**) showing positive GD2 antigen presentation on the surface of these cell types (representative flow of *n* = 4).

β1,4-N-acetylgalactosaminyltransferase (β4GANT*1*, GM2/GD2 synthase) is a key enzyme which is responsible for the synthesis of the glycosphingolipids GM2, GD2, and GA2. In a previous publication by Woo *et al*. [16], disialogangliosidase 2 (GD2) was identified as a GBM-specific antigen, in addition to a glycosphingolipid, CD90. As an initial screen, a variety of cell lines were assessed with respect to their relative expression of β4GANT*1* mRNA. As a reference, we used cervical cancer HeLa cells, which have been shown to express β4GANT*1* [16]. Cell lines assessed included the colorectal HT29 and lung cancer A549 lines, expressing less or equivalent amounts of β4GANT*1* as the HeLa controls. The GBM commercial and primary lines, A172, MZ-327 and MZ18 expressed little or no GD2 synthase compared to HeLa cells, while U251 or patient derived primary RN1 spheroid culture and JK2, or recurrent MZ-256 and MZ-304 lines expressed significantly higher levels of GD2 synthase (Figure 3A). Although the commercially available GBM cell line U251 expressed β4GANT*1*, it was of interest to the authors to assess patient-derived lines in this study. Expression of GD2 synthase patient-derived lines MZ-256, MZ-304 and RN1-spheroid was further confirmed by flow cytometry analysis (Figure 3B). Based on their high GD2 synthase expression levels these lines were chosen for further investigations.

### Characterisation of PLGA-Let-NPs and visualisation of anti-GD2-ch14.18/CHO-PLGA-Alexafluor647-NPs in glioblastoma-colorectal cancer cell co-culture

**Figure 4:**
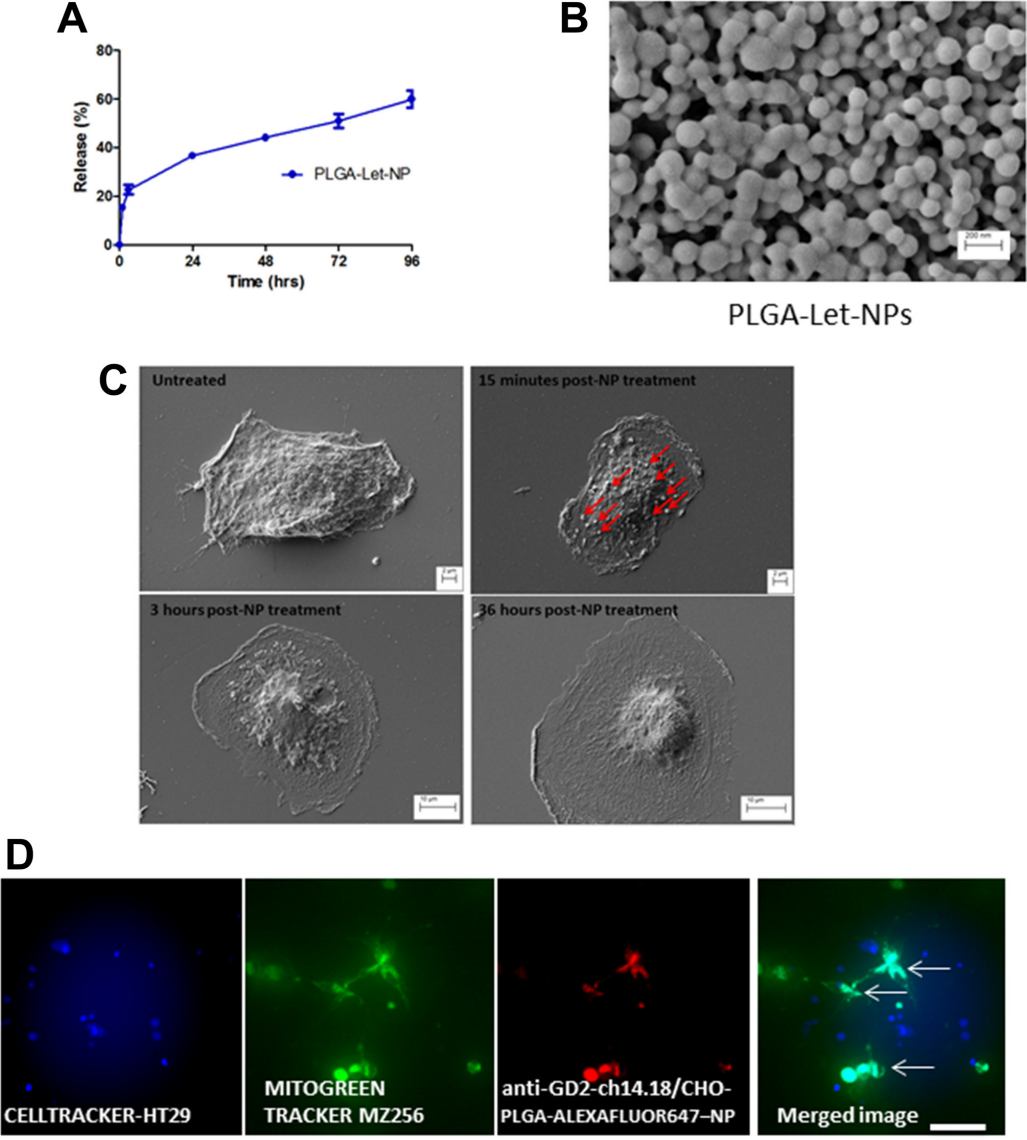
Characterisation of PLGA-Letrozole-NPs and anti-GD2-ch14.18/CHO-PLGA-NP mediated delivery of Letrozole exclusively to GBM cells in co-culture Nanoparticles were prepared from a PLGA polymer using a modified single emulsion evaporation method [32]. Size and zeta potential of each nanoparticle batch was measured in a Malvern Zetasizer. As evaluated through a content release study, 50% content release of PLGA-Let-NPs occurs 72 hours post-incubation in phosphate buffered saline (PBS) at 37°C (**A**). This was also the case in cell culture media (data not shown). SEM imaging displays PLGA-NPs at 50Kx maginification (**B**). In addition, SEM imaging (**C**) display the morphological changes which occur 15 minutes post-NP treatment with NPs evident on the cell surface (denoted by red arrows), 3 hours post-NP treatment and 36 hours later. (**D**) HT29 colorectal cancer cells were pre-incubated with Cell tracker Blue reagent while MZ-256 GBM cells were incubated with MitoGreen tracker prior to co-culture on glass bottom dishes. As shown, NP positive cells are represented as *Cyan* in the merged image of *Blue* Cell-tracker-HT29, *Green* Mitogreen-MZ-256 and *Red*-NP. Notably, NP encapsulation is noted exclusively in those cells who are also MitoGreen positive, that is, MZ-256 cells and not in HT29 (Blue) cells (Scale 50 μm).

PLGA is an FDA-approved biodegradable, physically strong and highly biocompatible polymer; especially suitable as a delivery vehicle for drugs, proteins and other macromolecules including DNA, RNA and peptides [28–30]. Reasons for its popularity among various available biodegradable polymers are its favourable degradation characteristics and its suitability for sustained drug delivery. Toxicological studies have designated PLGA as an extremely safe material for macroscopic and micro particle systems [31]. Nanoparticles were prepared from a PLGA polymer using a modified single emulsion evaporation method [32]. In order to characterize the synthesized nanoparticles, size and zeta potential of each nanoparticle batch was measured in a Malvern Zetasizer. Nanoparticles, which contained Letrozole, (PLGA-Let-NPs) were found to possess a mean size of 143.6 nm ± 27.84, with a mean zeta-potential of -21.58 mV ± 0.632 and an encapsulation efficiency of 82.22% + 5.77. Supplementary Figure 1 Nanoparticle imaging was carried out using scanning electron microscopy (SEM, Figure 4B). As evaluated through a content release study, there is an initial burst release of Letrozole within the first 12 hours, with 50% of content release noted after 50 hours of incubation in both cell culture media and phosphate buffered saline (PBS) at 37°C (Figure 4A). The anti-GD2-ch14.18/CHO antibody-NP conjugation protocol, modified from Kocbek *et al*. [33] was optimised through positive conjugation verified by rhodamine fluorescence resulting from anti-mouse-Rhodamine labelled secondary binding (data not shown). The morphological effects of anti-GD2-ch14.18/CHO-Let-NP treatment of MZ-304 GBM cells was evaluated by SEM imaging (Dr. Clodagh Dooley, AML, CRANN-TCD). NPs can be visualised 15 minutes post-NP addition in 4% paraformaldehyde fixed cells. These NPs are no longer evident on the cell surface after 3 hours due to internalisaton with the effects of Let-release within the GBM cells evident in Figure 4C. Data of control-NP treatment of GBM cells is displayed in Supplementary Figure 1.

### Specific delivery to GBM cells using anti-GD2-ch14.18/CHO conjugated PLGA nanoparticles

Specific cell delivery of potential therapeutics is a highly relevant issue, especially for diseases such as brain cancer where off-target drug delivery holds the potential to cause deleterious adverse effects and toxicity. To evaluate the potential of anti-GD2-ch14.18/CHO antibody to direct targeted delivery in GBM cells, PLGA-Alexafluor647-tagged-NPs were surface activated and coupled to anti-GD2-ch14.18/CHO antibody. HT29 colorectal cancer cells were pre-incubated with Cell tracker Blue reagent while MZ-256 GBM cells were incubated with MitoGreen tracker prior to co-culture on glass bottom dishes. As shown in Figure 4D, NP positive cells appear *Cyan* in the merged image of *Blue* Cell-tracker-HT29, *Green* Mitogreen-MZ-256 and *Red*-NP. Notably, NP encapsulation occurs exclusively in mitotically active MZ-256 cells as evidenced by their positivity for MitoGreen.

### Treatment of GBM cells with anti-GD2-ch14.18/CHO -PLGA-Let-NPs ± TMZ

**Figure 5:**
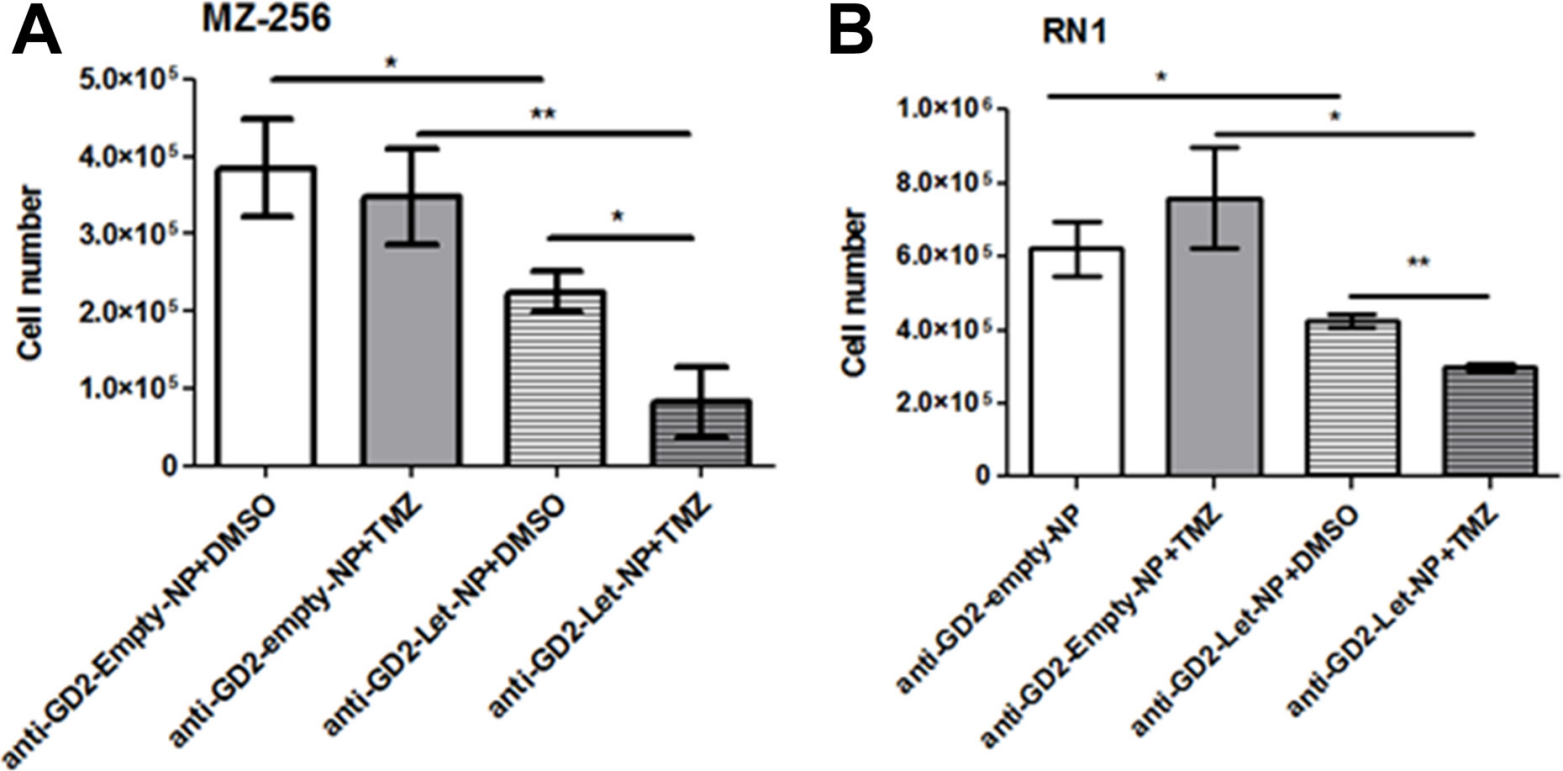
Treatment of GBM cells with anti-GD2-ch14.18/CHO-PLGA-Let-NPs ± **TMZ**. Glioblastoma cell lines MZ-304 (**A**) and RN1 (**B**) were treated with anti-GD2-ch14.18/CHO-PLGA-Let-NPs or the corresponding control anti-GD2-ch14.18/CHO-PLGA-Veh-NPs with or without temozolomide (150 μM)-supplemented cell media. Treatment of both cell lines with anti-GD2-ch14.18/CHO-PLGA-Let-NPs in combination with TMZ significantly reduced cell numbers when compared to anti-GD2-ch14.18/CHO-PLGA-Veh-NPs+TMZ or anti-GD2-ch14.18/CHO-PLGA-Let-NPs+DMSO treatments alone (*n* = 3, mean ±SEM, *p < 0.05, **p < 0.01, ***p < 0.001). Notably, for ease of labelling in the above figure anti-GD2-ch14.18/CHO-PLGA-NPs are referred to as anti-GD2-NPs.

The standard treatment regimen for newly diagnosed glioblastoma patients is based on the so-called Stupp protocol [4], involving fractionated radiotherapy (RT) in combination with temozolomide (TMZ). The addition of TMZ to RT results in a clinically significant survival benefit [9]. TMZ is administered with fractionated radiotherapy and consists of a dose of 75 mg/m^2^ per day, given from the first until the last day of RT, but not longer than 49 days. After a four week break up to six cycles of adjuvant TMZ at a dose of 150 mg/m^2^ follow. Based on the findings of Stupp *et al*. median survival rate was increased from 12.1 months with RT alone to 14.6 months with TMZ and RT treatment combination. The contribution of aromatase to chemoresistance has been noted for several other cancers [34]. In order to assess if this may also be the case for GBMs, glioblastoma cell lines MZ-304 and RN1 were treated with anti-GD2-ch14.18/CHO-PLGA-Let-NPs or the corresponding control NP containing DMSO vehicle (anti-GD2-ch14.18/CHO-PLGA-empty-NPs) with or without temozolomide (150 μM)-supplemented cell media. Notably, treatment of both cell lines with anti-GD2-ch14.18/CHO-PLGA-Let-NPs in combination with TMZ (Figure 5A and 5B) significantly reduced cell numbers when compared to anti-GD2-ch14.18/CHO-PLGA-empty-NPs+TMZ or anti-GD2-ch14.18/CHO-PLGA-Let-NPs+DMSO treatments alone (*p < 0.05, **p < 0.01, ***p < 0.001).

### Assessment of the role of miR-191 in aromatase inhibitor-induced reduction of GBM cell proliferation

**Figure 6:**
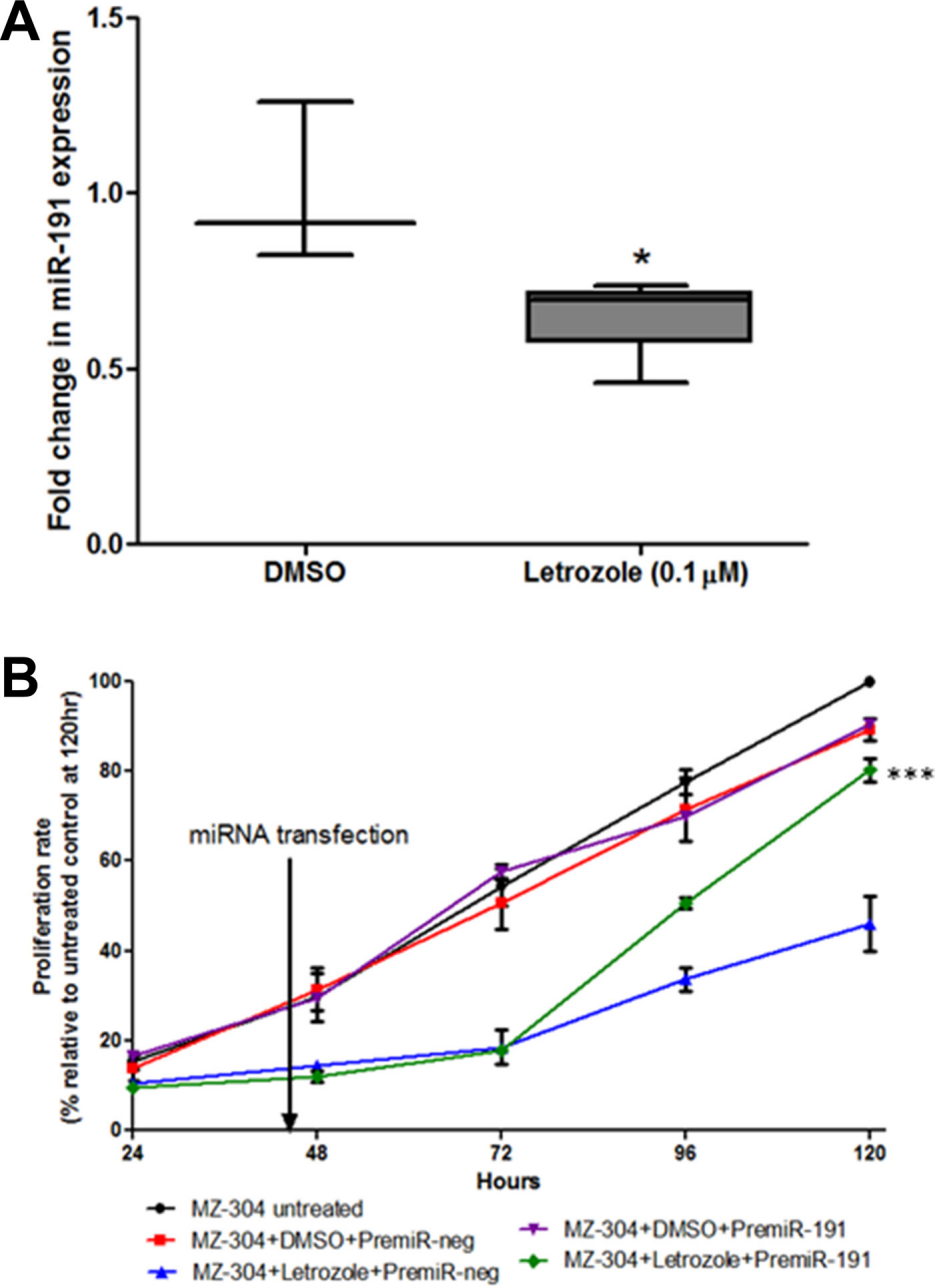
Assessment of miR-191contribution to GBM-letrozole treatment GBM cells (MZ-304) were treated with Letrozole (0.1 μM) and qPCR was carried out to assess the effect of aromatase inhibition on the oestrogen response microRNA, miR-191. Notably, Letrozole treatment resulted in a 34.46% ± 4.99 reduction in miR-191 expression relative to vehicle-treated controls (**A**), *n* = 3, mean ± SEM *p < 0.05). Therefore, the ability of reintroduced synthetics premiR-191 into Letrozole-treated GBM cells to rescue the reduced-proliferation phenotype was assessed over 120 hours (**B**). It was found that transient transfection of miR-191 Let-treated MZ-304 cells led to a restoration of ‘normal’ cell proliferation rates, similar to those noted in untreated and DMSO+premiRneg-treated MZ-304 cells (***p < 0.001, *n* = 3, mean ± SEM, Two way ANOVA).

The microRNA-191 (miR-191) has been implicated in several cancer types [35–38] showing an active role in cell proliferation, migration, chemoresistance and ultimately disease progression. As miR-191 is an estrogen-responsive microRNA [39], and its expression has been correlated to the aggressive mesenchymal subtype of GBM [40], it was of interest to evaluate if the treatment with Letrozole (0.1 μM) (Figure 2) alters miR-191 expression in glioma cells. Therefore, qPCR was carried out to assess the effect of aromatase inhibition on levels of miR-191. The results showed that Letrozole treatment results in a 34.46% ± 4.99 reduction in miR-191 expression relative to vehicle-treated controls (Figure 6A *p < 0.05). It was of interest, therefore, to assess if the reduction of GBM cell proliferation after Letrozole-treatment could be reversed by reintroduction of miR-191. As shown in Figure 6B, transient transfection of synthetic miR-191 into MZ-304, whose growth rate had been significant reduced by Letrozole treatment, led to a restoration of cell proliferation rates back to basal levels of untreated cells. Treatment with premiR-191 returned Letrozole-treated MZ-304 cells growth rate to that similar to untreated and DMSO+premiRneg-treated cells over a 120 hr period (***p < 0.001, *n* = 3, mean ± SEM, Two way ANOVA).

## DISCUSSION

Previous studies carried out by Dave *et al*. [41] investigated the efficacy of the aromatase inhibitor, Letrozole, on GBM cell growth *in vitro* and *in vivo* exclusively in the conventional human glioma cell lines and the rat glioma C6 cells. Several studies have shown that treatment response in established glioma cells differs greatly to that noted in patient-derived primary cultures [42, 43]. Therefore, it was of great interest to the authors to ascertain if aromatase inhibitors manifest an anti-tumor effect also in primary cultures derived from both primary and recurrent GBMs, and on patient-derived stem-like glioma cells. In addition, the possibility of selective targeting of glioma cells through anti-GD2-ch14.18/CHO conjugated PLGA nanoparticles as an approach to limiting off target effects was addressed.

Aromatase is a microsomal cytochrome P450 complex coded by the CYP19A1 gene [44]. Aromatase catalyses the irreversible conversion of androgens to oestrogens [13] with activity in several normal and cancer tissues, the latter including brain cancers [14, 45, 46] and this study. Inhibition of aromatase is promising for the treatment of hormone-dependent breast cancer, pathological alterations of ovarian and endometrial function and some benign disorders like gynecomastia associated with uncontrolled cell proliferation. As found in this study, the addition of the aromatase inhibitor, Letrozole, to patient-derived GBM cultures led to a significant reduction in proliferation, migration and gliomasphere forming capacity *in vitro*. Our findings are not only concordant with those reported in conventional glioma cell lines by Dave *et al*. [41] but also extend these previous findings to a more clinically relevant experimental models such as glioma stem cells by integrating cell type-specific targeting approach based on the utilization of surface biomarkers expressed in GBM cells.

In the context of breast cancer, an estrogen-dependent cancer, where the use of aromatase inhibitors has been highly researched [47–51], deleterious effects of non-specific delivery of Letrozole includes osteoporosis leading to increased bone fractures, joint and muscle pain, menopausal symptoms, depression, cardiac problems, increased cholesterol and increased blood pressure were shown. In this regard, specific delivery of aromatase-inhibitors exclusively to target cancer cells using antibody conjugated biodegradable nanoparticles provides a promising approach for increasing drug efficacy and reducing therapeutic side effects. With respect to GBM, recent studies have shown that several antigens including cluster of differentiation 90 (CD90, Thy-1) and GD2 disialoganglioside are frequently overexpressed in glioblastoma cells [52]. GD2 disialoganglioside is a sialic-containing glycosphingolipid, also shown to be overexpressed in neuroblastoma with the GD2 specific antibody anti-GD2-ch14.18/CHO being used in several neuroblastoma clinical trials [20, 21]. We also reported previously that the anti-GD2-ch14.18/CHO antibody was successfully used for nanoparticle-mediated specific delivery of microRNAs to neuroblastoma tumors *in vivo* [53].

β-1,4 N-acetylgalactosaminyltransferase 1 encoded by the β4GALNT1 gene is an enzyme responsible for the biosynthesis of G(M2) and GD2 glycosphingolipids [54]. As depicted in Figure 3A, β4GALNT1 transcript expression was assessed as an indicator of GD2 expression across a panel of human cancer cell lines including conventional cell lines derived from GBM and patient-derived primary cultures of first diagnosed or recurrent GBM. Our results reveal the benefits of a combinatorial approach integrating nanoparticles-based delivery of the aromatase inhibitor Letrozole with the selective antibody-mediated cell targeting cell-specific recognition.

Although Letrozole has shown to be capable of blood-brain barrier (B-BB) transition in rat models of glioblastoma with high uptake rates into the tumor itself [55], specific delivery through encapsulation within biodegradable nanoparticles is a promising avenue of research and may provide the means to minimize the adverse effects of current chemotherapeutics in use for GBM treatment. The intricate composition of the B-BB ensures the highly regulated and restricted transport of molecules with limitations further imposed by electrical resistance across B-BB endothelia [56]. Differences in nanoparticle material, size and shape and receptor (over)expression makes it difficult to define optimal criteria for B-BB diffusions; however, passive transport typically occurs for lipophilic molecules below 400Da and through several studies it has been shown that 80–200 nm nanoparticle size range is optimal for tumour inclusion and effect [57]. In addition, conjugation of a GD2 specific antibody to the surface of activated Letrozole-encapsulated PLGA-nanoparticles renders GBM cell specificity. It should be noted that antibody conjugation significantly alters the cell surface of the nanoparticles and nanoparticle size as shown in Supplementary Table 2, presumably due to anti-GD2-ch14.18/CHO antibody-antibody aggregation. Such an aggregation causes the nanoparticles to behave like larger particles [58] and may affect systemic delivery for therapeutic application in the brain. However, evaluation of potential therapeutics through direct intratumoral injection including nanoparticle delivery to *in vivo* models of GBM [59, 60] or at the point of patient tumor resection or biopsy [61–65] in several studies indicates that such systemic transport challenges can be circumvented to facilitate therapeutic delivery in a clinical setting.

Nanoparticle formulation for this study was based on the protocol by Jana *et al*. (2014) which used a single emulsion and evaporation method for letrozole encapsulation and formulation of PLGA nanoparticles [32]. Similar to the aforementioned protocol, this study resulted in the formulation of PLGA-NPs less than 150 nm with an encapsulation efficiency of over 80%. Notably, however, anti-GD2-ch14.18/CHO antibody conjugation resulted in a dramatic increase in NP size (>360 nm) which although would affect B-BB penetrance and cellular diffusion; had no observable bearing on GBM cellular uptake as a result of direct administration in this study due to the pharmacokinetics of GD2 antigen-antibody endocytosis on the GBM cell surface. Although these sized NPs would have a significant effect on B-BB penetrance, treatment protocols for recurrent GBM tumour resection provides an opportunity for direct therapeutic administration to the brain tumour cavity; thereby circumventing the B-BB.

In order to assess whether Letrozole administration improves chemoresponse to TMZ in GBM cells, anti-GD2-ch14.18/CHO-PLGA-Let-NP in combination with temozolomide (TMZ, 150 μM) treatment in GBM cells *in vitro* was assessed. The findings show that the anti-cancer effects elicited by the Letrozole-nanoparticles in GBM cells attenuate TMZ-induced reduction in cell numbers over 72 hours. Although these findings need to be assessed in an orthotopic model of glioblastoma, this *in vitro* study highlights the successful encapsulation and delivery of functional Letrozole exclusively to GBM cells.

In an attempt to understand the mechanism through which Letrozole elicits its anti-tumor effect in GBM cells, the authors evaluated the expression levels of the estrogen-responsive microRNA-191 (miR-191) post-Letrozole treatments. MiR-191 has been shown to be a prognostic indicator of poor survival rates in GBM patients [40], indicating a potential role for hormonal control of GBM pathogenesis. Oestrogen is synthesised, catalysed by aromatase, and subsequently binds to the estrogen receptor (ER) alpha/beta subunits which dynamically interact with the estrogen responsive elements (ERE) in the promoter region of miR-191 [39], increasing expression and driving proliferation, migration and chemoresistance in several cancer types [54, 66–68]. It is hypothesized that aromatase inhibition through Letrozole treatment reduces oestrogen-mediated binding of the ER to the ERE and therefore reduces miR-191 expression. In order to assess this hypothesis the authors investigated the ability of miR-191 to rescue the reduced proliferative phenotype induced by Letrozole in GBM cells and found that reintroduction of synthetic premicroRNA-191 led to significant restoration of ‘normal’ growth patterns of the GBM cell *in vitro*. In this regard, it may be of interest to evaluate the therapeutic merit of miR-191 as a potential anti-tumor molecule for future studies.

Glioblastoma is a highly aggressive brain cancer with an extremely poor prognostic outcome despite intensive treatment regimes. With an average five year survival rate of less than 3%, this dismal prognosis necessitates exploration of novel therapeutics and drug delivery systems such as those detailed in this study. The identification of new mechanism-based therapeutic targets through repurposing of currently approved clinical drugs, and the use of alternative drug-delivery strategies such as tumour-specific mediated delivery of effective anti-cancer drugs, improves upon this bleak outlook, and is an important agenda with respect to GBM research.

## MATERIALS AND METHODS

### Cell lines and patient-derived tumor samples

For all *in vitro* experiments glioblastoma cell lines derived from patient GBM tumor biopsies and from other cancer types such as colorectal (HT29), lung (A549) and cervical (HeLa) were used. A172 and U251 are commercially available GBM cell lines which, in addition to the MZ-304, MZ-327 and MZ-256 cell lines [69, 70] were grown as a monolayer in DMEM media (BioWhittaker^®^) with 10% heat-inactivated fetal calf serum (FCS; GIBCO), 100U/mL penicillin (Sigma-Aldrich^®^), and 100 μg/mL streptomycin (Sigma-Aldrich^®^). Stem-like glioma initiating cell lines (GICs) established from human GBMs were previously described [71, 72]. Low-passage primary GBM patient-derived lines, RN1 and JK2, were kindly donated by the Brain Cancer Research Unit, QIMR Berghofer Medical Research Institute, Australia. The cells were grown as a monolayer (adherent condition) in Serum-Free Human NSC Culture Medium, as previously described [73], supplemented with β-FGF and EGF (10 ng/ml and 20 ng/ml respectively) on 1% matrigel-coated flasks or plates [73] or as a gliomasphere culture in the absence of matrigel and presence of heparin (10 ng/μl, Sigma Aldrich). These RN1 cells stably express luciferase if required. All cells were maintained in a humidified incubator at 37°C with 5% CO2. Patient clinical data pertaining to the GBM cell lines used is presented in Supplementary Table 1.

### Protein extraction

Glioblastoma cells were washed with 1x Phosphate buffered saline (PBS) (BioWhittaker^®^), trypsinised (Trypsin:EDTA, Sigma-Aldrich^®^) and centrifuged at 1,200 rpm for 4 minutes. The supernatant was removed and protein was extracted from the resulting pellet in Protease inhibitor (1:100, Sigma-Aldrich^®^) and RIPA buffer (1M Tis pH 7.4, 5M NaCl, 1% NP-40, 50 mM NaF, 0.5M EDTA, 0.1% SDS, 0.5% Na-deoxycholate). Resuspended cells in RIPA were left on ice for 20 minutes before centrifugation at 4°C at 12,000 rpm for 10 minutes. Supernatant was isolated, quantified using a BCA™ Protein Assay kit (Thermo Fisher Scientific) and stored at –20°C.

### Western blot

Western blot analysis was performed on lysates prepared as outlined previously, or lysates provided from patient-derived glioblastoma xenografts (G46, G59, G64, G76, G79, G80, G84, G85 and G91) by Mayo Clinic Brain Tumor SPORE [24–27]. Clinical data related to these patient-derived samples are detailed in Supplementary Table 1. Western blots were carried out using 4–10% gradient pre-cast gels (Thermo Fisher Scientific) and Tris-HEPES-SDS running buffer (0.1M Tris, 0.1M HEPES, 3 mM SDS, pH 8) (Thermo Fisher Scientific). Protein samples were mixed with 1x Lamelli buffer containing 1,4-dithiothreitol (DTT) (31.5 mM Tris-HCL, pH 6.8; 10% glycerol, 1% SDS, 0.005% Bromophenol blue, 50 mM DTT). Gels were run at 80V for 45 minutes, followed by transfer onto nitrocellulose membranes (GE Healthcare Life Sciences) at 40V for 90 minutes, in transfer buffer (48 mM Tris, 30 mM Glycine, 0.037% SDS, 2.5% methanol pH8.3). Blocking buffer (10%-milk-TBST, Marvel, (Tris, Tween20^®^) Sigma-Aldrich^®^) was used to block nonspecific sites on the membrane at 4°C overnight. Membranes were incubated with primary anti-P450 antibody (Abcam 1:250 in TBST) at room temperature for 2 hours and washed with TBST. Secondary anti-rat IgG antibody (Abcam, 1:2000 in TBST) was then added for two hours, followed by further washes with TBST. For control loading, β-actin antibody (Abcam, 1:5000 in TBST) was added for one hour at room temperature. Membranes were washed with TBST and secondary anti-mouse IgG antibody (Abcam, 1:2000 in TBST) was added for one hour, followed by further washes with TBST. All membranes were developed using the Pierce™ ECL Plus Western Blotting Substrate and the images were assessed on a LAS Imager4000.

### RNA extraction, cDNA synthesis and qPCR

RNeasy^®^ Mini kit (QIAGEN) was used to extract RNA from all cell lines. cDNA synthesis was performed using the High capacity cDNA Reverse Transcription Kit (Applied Biosystems™) according to the manufacturer's protocol. Aromatase mRNA transcript (*CYP19A1*) expression was assessed in several cell lines relative to β-actin by qPCR, using a *CYP19A1*-specific Taqman probe (Applied Biosciences) and β-actin as an endogenous gene control. GD2 expression was assessed using qPCR of the β1,4-N-acetylgalactosaminyltransferase (β4GALNT1, GM2/GD2 synthase) gene using a specific Taqman probe. Detection of mRNA transcript was carried out on a StepOne Plus qPCR machine under the following parameters 50°C for 2 minutes, 95°C for 10 minutes, followed by 40 cycles of denaturation at 95°C for 15 seconds and 60°C for 60 seconds. Expression levels were calculated based on the delta delta CT (ΔΔC_T_) method.

### Fluorescence activated sorting analysis

Log phase GBM cells (2 × 10^6^, MZ-304, MZ-256 and RN1-spheroid) were harvested and stained with GD2-FITC antibody (Santa Cruz, sc-53831). Cells were then analysed by a FACS Calibur (BD Bioscience).

### Cell proliferation assay

GBM cells (MZ-304, MZ-256 and RN1) were seeded at 1 × 10^5^cells/well in a 6-well plate (Sarstedt AG&Co). After 24 hours, media was removed and replaced by either vehicle control (DMSO)-media (Sigma-Aldrich^®^) or Aromatase inhibitor (AI) Letrozole (Sigma-Aldrich^®^) supplemented media (0.1 μM). Cells were then allowed to grow for a further 72 hours and cell numbers were determined through trypan-blue exclusion.

### 2-Dimensional migration assay (Wound healing/scratch assay)

In order to test the migratory potential of cells post-treatment with DMSO as a control or Letrozole (Let, 0.1 μM), GBM cells (MZ-304, MZ-256 and RN1) were seeded at 1x10^4^cells per side of a 2D Migration chamber (Ibidi GmbH) and allowed to grow to confluency. After 24 hours, the separator was detached using sterile tweezers, DMSO or Letrozole (0.1 μM) supplemented media was added and initial ‘wound’ images were taken. Cells were allowed to grow for 24 hours and the extent of cell movement was determined by taking an image after the incubation time, relative to the DMSO-treated control.

### PLGA-Let-Nanoparticle (PLGA-Let-NP) formulation

Letrozole (Let) loading of PLGA nanoparticles was carried out according to the modified single-emulsion evaporation method outlined by Jana *et al*. [32], modified from [74]. Briefly, PLGA 50:50 (Resomer 503H 200 mg/ml, Sigma Aldrich) polymer was dissolved in an organic phase of chloroform. Letrozole (10 mg) or control DMSO was dissolved in an acetone - dichloromethane (1.5 ml 1:2 v/v) mixture. The PLGA-letrozole mixture was then emulsified with an aqueous phase composed of 2% polyvinyl alcohol (PVA) solution in water (4.5 ml) by sonication using a micro tip probe sonicator at an output of 50W for 30s in an ice bath. The organic solvents were then rapidly removed by evaporation at 37°C leaving behind colloidal suspension of PLGA NPs containing Letrozole (PLGA-Let-NPs) or a DMSO control (PLGA-empty-NPs) in water, which was then pelleted using an ultracentrifuge at 4°C at 30,000 rpm for 15 minutes. Each pellet was washed with 1.13% w/v NaCl solution to keep the particles small. This was carried out three times. The resulting pellet was resuspended in molecular grade water and placed at –80°C for at least 30 minutes. This was then lyophilized overnight and the resulting powder was weighed to determine yield.

### Nanoparticle characterisation

Each nanoparticle preparation was weighed in a sterile Eppendorf tube and resuspended in molecular grade water. This suspension was then placed on a Zeta cell and measured in a Malvern Zetasizer to determine size and zeta potential of each nanoparticle batch. To determine encapsulation efficiency, an aliquot of PLGA-Let-NPs (1 mg) was resuspended in acetone and subjected to centrifugation at 20,000 rpm at 25°C for 30 min. The amount of drug present in the pellet was determined using a standard curve, constructed using varying Letrozole concentrations (0–10 μg/ml in acetone, R^2^ = 0.9778) versus absorbance at 238 nm [75]. Encapsulation efficiency was calculated using the following formula:

$$\text{Encapsulation efficiency}(\%)=\frac{\text{Actual drug loading *(mass) in 1 mg samples }\times 100}{\text{Theoretical drug loading in a 1 mg sample}}$$

Letrozole release was calculated by resuspending lyophilized PLGA–Let-NPs (1 mg) in PBS (1.2 ml pH 7.4) and the solution was divided in 6 microfuge tubes (200 μl each), maintained at 37°C in a thermo-stable water bath. After an appropriate time period (1 hr, 24 hr, 48 hr, 72 hr, 96 hr and 120 hr) the solution was centrifuged at 3000 rpm. Absorbance of this sample supernatant was measured at 238 nm. Concentration of the released Let was then calculated using the standard curve of the respective drug (0–10 μg/ml, R^2^ = 0.9966). The percentage of Let released was determined from the following equation:

$$\text{Release at time t}(\%)=\frac{\text{Mass for each sample containing }0.17\text{ mg NPs}\times 100}{\text{Mass of encapsulation efficiency in }.0.17\text{ mg of NPs}}$$

*Note: For release, Starting mass of NPs was 1 mg, divided across 6 eppendorfs (therefore there was 0.17 mg in each tube containing 200 μl)*.

### Conjugation of NPs to anti-GD2- ch14.18/CHO antibody

Based on the antibody conjugation to nanoparticle protocol used by Kocbek *et al*. [33], PLGA-NPs were resuspended in sterile filtered MES buffer (4-morpholinoethanesulfonic acid, pH 5.5) (Sigma-Aldrich^®^) and shaken at room temperature for 1 hour. After centrifugation at 4°C at 13,000 rpm for 20 minutes the MES buffer was discarded, giving a pellet of activated NPs. EDC-(N-[3-Dimethylaminopropyl]-N-ethylcarbodiimide hydrochloride) NHS-(N-Hydroxysuccinimide) (Sigma-Aldrich^®^) 1xPBS-mix at a 8:8:1 ratio was prepared. Both, the EDC-NHS-1xPBS-mix and anti-GD2-ch14.18/CHO antibody were added to the activated NPs, which were then shaken at room temperature overnight. The anti-GD2-ch14.18/CHO antibody was made available by a European funding effort by charities and commissioned by SIOPEN. This project is now further developed by APEIRON Biologics. A centrifugation at 4°C at 13,000 rpm for 20 minutes followed. The washing steps included a resuspension of the pellet in 1xPBS (0.01M; pH 7.15) and a further centrifugation as previously, which was repeated twice. The resulting pellet was resuspended in nuclease-free water and placed at -80°C for at least 30 minutes. NPs were then lyophilized overnight and the resulting powder was weighed to determine yield.

### Assessment of GBM-specificity using anti-GD2-ch14.18/CHO-PLGA-Alexafluor647-NPs in a glioblastoma-colon cancer cell co-culture

In order to assess the ability of anti-GD2-ch14.18/CHO antibody to direct nanoparticle-mediated drug delivery to GBM cells, a co-culture system was generated involving the GBM cell line MZ-256 and the colorectal cell line (HT29). HT29 colorectal cancer cells (1 × 10^5^) were pre-incubated with Cell tracker Blue reagent (25 μM) while MZ-256 GBM cells (1 × 10^5^) were incubated with MitoGreen tracker (50 nM) prior to co-culture on glass bottom dishes in DMEM-10%FCS media. To enable visualisation of nanoparticle delivery PLGA-nanoparticles were generated which encapsulated an Alexafluor647 fluorophore. This fluorescent tag is only detectable after PLGA-NP degradation and content release. Anti-GD2-ch14.18/CHO-PLGA-Alexafluoro647-NPs were synthesized and added for a 6 hour period, removed and cells were assessed by fluorescent microscopy after 96 hours.

### Treatment of GBM cells with anti-GD2-ch14.18/CHO-PLGA-Let-NPs and TMZ

Based upon encapsulation efficiency and release time calculations, MZ-304 and RN1 glioblastoma cells were treated with anti-GD2-ch14.18/CHO-PLGA-Let-NPs or the corresponding DMSO vehicle control nanoparticle (anti-GD2-ch14.18/CHO-PLGA-empty-NPs). GBM cells (MZ-256) were seeded at 1 × 10^4^cells/well in a 6-well plate (Sarstedt AG&Co) and were allowed to grow. After 24 hours anti-GD2-ch14.18/CHO-PLGA-Let-NPs were resuspended in sterile media/PBS at a concentration of 1 mg/ml. Based on the determined average encapsulation efficiency of 82%, 8.2 mg (2.87 μM) of Letrozole was calculated to be present per mg of NP resuspended in 1 ml of media. Accordingly, an appropriate amount of resuspended NP was added to the cell culture media to ensure a final concentration of 0.1 μM of Letrozole. NPs were removed after 6 hours and temozolomide (TMZ) or DMSO vehicle control-supplemented (150 μM) cell media was used. Cells were then allowed to grow for a further 72 hours and cell numbers were assessed.

### PremiR-191 rescue experiment of Letrozole-treated GBM (MZ-304) cells

GBM cells (MZ-304) were transiently transfected with premiR-191 (30 nM, Applied Biosystems) using siPORT NeoFx transfection reagent (Applied Biosystems). In brief, MZ-304 cells were seeded (1 × 10^4^ cells) into a T25 flask in normal cell culture media. After 6 hours, to allow adherence, media was replaced with control (DMSO) or Letrozole (0.1 μM)-supplemented media and proliferation rates were determined using trypan blue exclusion at various time points. After 40 hours, MZ-304 ± Letrozole cells were transiently reverse transfected with control negative (premiR-neg) or synthetic precursor miR-191 (premiR-191) and returned to assess subsequent growth.

### Statistical analysis

For comparison of multiple unpaired samples, OneWay ANOVA was used with Kruskal-Wallis H Test; while for direct comparison of two unpaired biological samples, Mann Whitney u test was used in Prism-Graph Pad. All assays were carried out with technical repeats of *n* = 2–3 and in biological triplicate and the values presented as mean ± SEM. *p < 0.05; **p < 0.01; ***p < 0.001.

## SUPPLEMENTARY MATERIAL FIGURE AND TABLES


