# Supplementary material for: Anti-GD2-ch14.18/CHO coated nanoparticles mediate glioblastoma (GBM)-specific delivery of the aromatase inhibitor, Letrozole, reducing proliferation, migration and chemoresistance in patient-derived GBM tumor cells
Source: Oncotarget. 2017 Feb 3;8(10):16605–20. doi: 10.18632/oncotarget.15073 (PMC5369988; doi:10.18632/oncotarget.15073)
Supplement: Supplementary file 1 [file oncotarget-08-16605-s001.pdf]

Anti-GD2-ch14.18/CHO coated nanoparticles mediate glioblastoma (GBM)-specific delivery of the aromatase inhibitor, Letrozole, reducing proliferation, migration and chemoresistance in patient-derived GBM tumor cells

Supplementary Materials

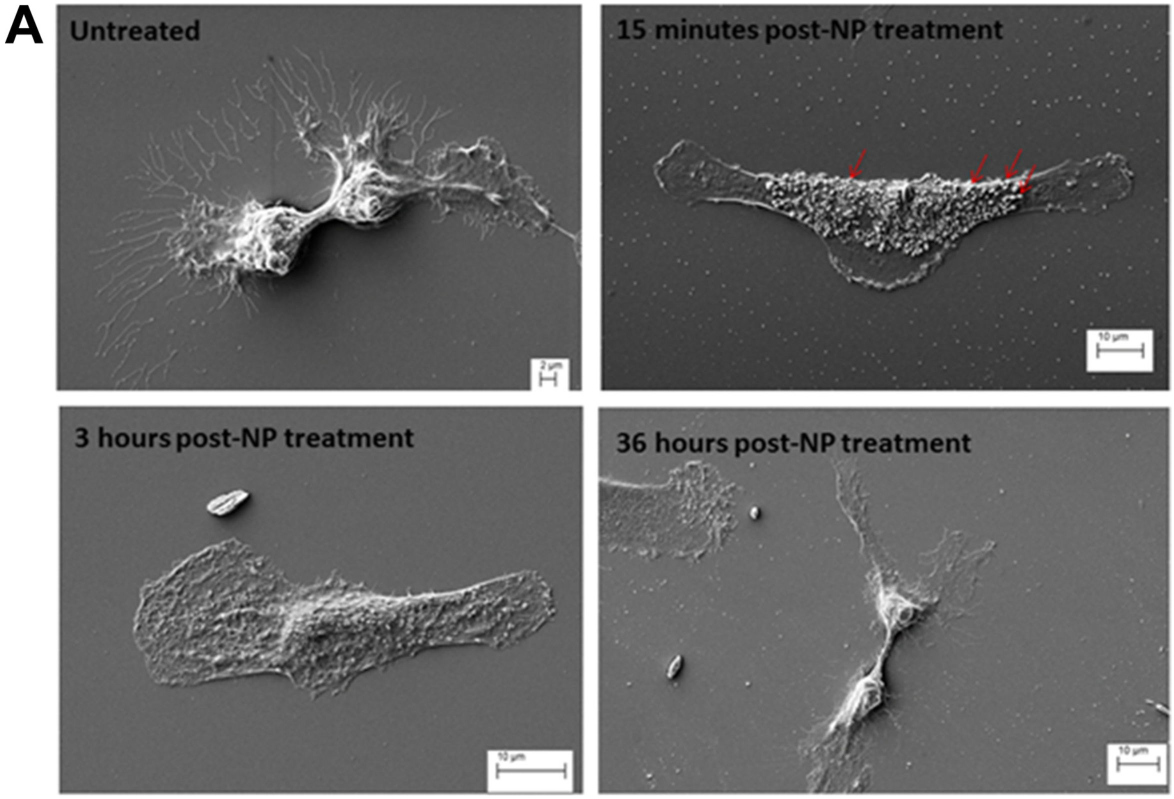

Supplementary Figure 1: Uncoated-Let-NP control experiment. SEM imaging displays the fact that there are no maintained observable morphological changes 36 hours post-NP treatment with uncoated-Letrozole loaded-NPs. NPs are evident on the GBM cell surface (denoted by red arrows) and cell morphology is altered after initial NP addition, however the significant and sustained cell modification which is noted in Figure 4C is not recapitulated in the control NP experiment; suggesting that the effect is due to the introduction of Letrozole within the cell itself.

Supplementary Table 1: GBM tumour-derived cell line characteristics

Sample	Subject diagnosis	GBM Sub type	*MGMT* status	Sex	Age (years)
**G46**	R	M	Met	M	56
**G59**	P	M	Met	F	83
**G64**	R	P	Un	F	64
**G75**	P	*	Un	F	62
**G76**	R	C	Met	M	38
**G79**	R	M	Un	F	71
**G80**	P	P	Un	M	49
**G84**	P	C	Met	F	49
**G85**	P	P	Met	M	78
**G91**	P	C	Un	M	62
**MZ-304**	R	*	Un	*	*
**MZ-327**	P	*	Un	*	*
**MZ-256**	R	*	Un	*	*
**RN1**	P	C	Un	M	56

Summary of clinical characteristics of patient-derived glioblastoma xenografts (G46, G59, G64, G76, G79, G80, G84, G85 and G91) by the Mayo Clinic Brain Tumor SPORE, in addition to primary (MZ-327 and RN1) and recurrent (MZ-256 and MZ-304) GBM patient-derived cell lines.

P- Primary GBM *R-Recurrent GBM*
M- Mesenchymal subtype *P- Proneural subtype* *C-Classical subtype*
Un- unmethylated MGMT status *Met- methylated MGMT status*
** Information unknown at the time of publication*

Supplementary Table 2: Nanoparticle characterisation

	Average Size (nm)	Encapsulation efficiency (%)	Charge (mV)
PLGA-Let-NP	143.6 + 27.84	63.0667 + 11.64	–21.58 + 0.632
PLGA-Veh-NP	147.6 + 21.06	62.567 + 7.194	–27.067 + 3.35
AntiGD2-Let-NP	356.3 + 18.34		–10.498 + 2.89
AntiGD2-Veh-NP	301.4 + 23.41		–11.027 + 3.487

Summary table of characterization of synthesized nanoparticles, uncoated (PLGA-NP) and anti-GD2-ch14.18/CHO coated (anti-GD2-ch14.18/CHO-PLGA-NPs) nanoparticles. Average sizing, encapsulation efficiency and charge is included.
